# A review of landmark studies on maintenance immunosuppressive regimens in kidney transplantation

**DOI:** 10.2478/abm-2024-0015

**Published:** 2024-06-28

**Authors:** Suwasin Udomkarnjananun, Maaike R. Schagen, Dennis A. Hesselink

**Affiliations:** Division of Nephrology, Department of Medicine, Faculty of Medicine, Chulalongkorn University and King Chulalongkorn Memorial Hospital, Thai Red Cross Society, Bangkok 10330, Thailand; Excellence Center for Solid Organ Transplantation, King Chulalongkorn Memorial Hospital, Thai Red Cross Society, Bangkok 10330, Thailand; Renal Immunology and Transplantation Research Unit, Faculty of Medicine, Chulalongkorn University, Bangkok 10330, Thailand; Center of Excellence on Translational Research in Inflammation and Immunology (CETRII), Department of Microbiology, Chulalongkorn University, Bangkok 10330, Thailand; Division of Nephrology and Transplantation, Department of Internal Medicine, Erasmus MC Transplant Institute, University Medical Center Rotterdam, Rotterdam 3000, The Netherlands

**Keywords:** calcineurin inhibitor nephrotoxicity, clinical trials, immunosuppression, kidney transplantation, maintenance regimen

## Abstract

Immunosuppressive medications play a pivotal role in kidney transplantation, and the calcineurin inhibitors (CNIs), including cyclosporine A (CsA) and tacrolimus (TAC), are considered as the backbone of maintenance immunosuppressive regimens. Since the introduction of CNIs in kidney transplantation, the incidence of acute rejection has decreased, and allograft survival has improved significantly. However, CNI nephrotoxicity has been a major concern, believed to heavily impact long-term allograft survival and function. To address this concern, several CNI-sparing regimens were developed and studied in randomized, controlled, clinical trials, aiming to reduce CNI exposure and preserve long-term allograft function. However, more recent information has revealed that CNI nephrotoxicity is not the primary cause of late allograft failure, and its histopathology is neither specific nor pathognomonic. In this review, we discuss the historical development of maintenance immunosuppressive regimens in kidney transplantation, covering the early era of transplantation, the CNI-sparing era, and the current era where the alloimmune response, rather than CNI nephrotoxicity, appears to be the major contributor to late allograft failure. Our goal is to provide a chronological overview of the development of maintenance immunosuppressive regimens and summarize the most recent information for clinicians caring for kidney transplant recipients (KTRs).

Since the first successful kidney transplantation in 1954, kidney transplantation has become the standard treatment for patients with end-stage renal disease (ESRD), offering significant benefits in terms of both patient survival and quality of life when compared with dialysis [1, 2]. The progress in kidney transplantation has been greatly facilitated by advances in various aspects of the procedure, including pre-transplant evaluation of donor and recipient, surgical techniques, tissue typing and human leukocyte antigen (HLA) matching, and post-transplant care [3, 4]. However, the most impactful advance in kidney and other forms of solid organ transplantation has been the development of immunosuppression [5, 6]. The introduction of the calcineurin inhibitors (CNIs) cyclosporine A (CsA) and later, tacrolimus (TAC), along with mycophenolic acid (MPA), has significantly reduced the acute allograft rejection rate to <15% and has improved kidney allograft survival to over 95% at 1 year after transplantation [6]. Nevertheless, the use of CNIs in earlier eras, when higher pre-dose concentrations (C_0_) were targeted, was limited by their nephrotoxic effects [7], [8], [9], [10].

In this review article, we aim to provide a brief historical perspective and an update on the landmark randomized, controlled, clinical trials (RCTs) that have significantly influenced our clinical approach to maintenance immunosuppression in kidney transplantation.

## The era of CNI and MPA

The concept of rejection in organ transplantation is grounded in the understanding of alloantigen recognition and sensitization [11]. Non-self HLA antigens on the allograft are primarily recognized by the recipient's T cells and subsequently by B cells, leading to T cell-mediated rejection and antibody-mediated rejection (ABMR), respectively [12]. Immunosuppressive medications have been utilized since the early era of solid organ transplantation to prevent transplant rejection. However, their use necessitates a delicate balance with the risk of adverse effects, notably infections, malignancies, and cardiovascular complications [13].

In the 1960s, azathioprine (AZA) and corticosteroids made up the standard immunosuppressive regimen for the prevention of acute rejection after kidney transplantation [14]. However, allograft survival was poor, and the acute rejection rate was as high as 60% in the first year after transplantation [2, 14]. In 1983, 2 landmark RCTs regarding the use of CsA in kidney transplantation were published in the New England Journal of Medicine and Lancet [15, 16]. A study conducted by the Canadian Multicentre Transplant Study Group compared kidney transplant recipients (KTRs) who received AZA vs. CsA, both in combination with prednisolone as part of their maintenance immunosuppressive regimen. The study found that the CsA group (n = 103) had significantly better allograft survival at 1 year compared with the AZA group (n = 107), with rates of 80% and 60%, respectively [15]. Similarly, results from the European Multicentre Trial Group demonstrated superior 1-year kidney allograft survival in KTR treated with CsA monotherapy (n = 117) compared with those who received AZA and prednisolone (n = 115), with survival rates of 72% and 55%, respectively [16]. These 2 RCTs were pivotal in integrating CsA into the standard maintenance immunosuppressive regimen following kidney transplantation.

Initially, CsA was prescribed in a fixed dose regimen of 5 mg/kg/d, which resulted in nephrotoxicity and poor kidney allograft function. Subsequent RCTs explored the use of reduced-dose CsA in combination with AZA and prednisolone. This approach aimed to mitigate the dose-dependent nephrotoxicity of CsA, while maintaining overall immunosuppressive efficacy. Notably, when administered at a lower dose of 2 mg/kg/d, as part of a triple therapeutic regimen, this protocol significantly improved kidney allograft function [17]. Conversely, KTR who received higher-dose CsA monotherapy experienced more adverse effects, particularly nephrotoxicity and gum hypertrophy [17]. As a result, the triple immunosuppressive regimen consisting of CsA, AZA, and prednisolone became the standard in the late 1980s. Around the same period, therapeutic drug monitoring (TDM) of CsA gained significant attention, marking CsA as the first immunosuppressive drug in solid organ transplantation to demonstrate a concentration–effect relationship [14, 18]. A higher concentration of CsA in the blood (initially in plasma, later whole blood) was demonstrated to be associated with a lower incidence of acute rejection but an increased likelihood of developing nephrotoxicity [19], [20], [21], [22]. The target concentration of CsA, determined using either the pre-dose (C_0_) or 2-h post-dose concentration (C_2_), serves as a surrogate for the total exposure measured by the area-under the concentration vs. time curve (AUC) [14, 18, 23]. Evidence indicates that utilizing C_2_, as opposed to C_0_, serves as a superior surrogate single concentration timepoint correlated with AUC. Comparative studies have shown that C_2_ is a more accurate predictor of kidney allograft loss [24], kidney function [25], [26], [27], and allograft rejection [27], [28], [29], [30], as extensively reviewed in dedicated articles on TDM [30], [31], [32], [33].

Not long after the incorporation of CsA into the standard AZA and corticosteroid immunosuppressive regimen for KTR, mycophenolate mofetil (MMF), a prodrug of MPA, demonstrated additional advantages when added to CsA-based regimens. A study, conducted by the European Mycophenolate Mofetil Cooperative Study Group, revealed that the addition of MMF to the CsA/prednisolone regimen resulted in a remarkable 60%–70% reduction in acute rejection episodes compared with a placebo [34]. This efficacy of MMF was further validated in another RCT that compared MMF with AZA in KTR who were receiving CsA and prednisolone. The MMF group exhibited a 16% lower incidence of either biopsy-proven rejection or treatment failure within 6 months compared with the AZA group [35]. Importantly, a pooled analysis of 3 phase III multi-center RCTs (the 3 pivotal trials) conducted in the US, Canada, Europe, and Australia, showed that the use of MMF compared with AZA, in the regimen containing CsA and corticosteroids, significantly reduced the incidence of acute rejection and resulted in better allograft function at 12 months [36]. It is worth noting that in another RCT, conducted in a more modern era, the use of a microemulsion form of CsA rather than the oil-based CsA, did not show a difference in terms of acute rejection and allograft survival when comparing MMF to AZA [37, 38]. The microemulsion form of CsA has more consistent exposure of CsA than the CsA form used in the older RCTs (including the 3 pivotal studies). A meta-analysis has confirmed the superiority of MPA over AZA in terms of graft survival and prevention of acute rejection [39]. As a result, MPA has replaced AZA and has become the primary antimetabolite used in the maintenance regimen of kidney transplantation in conjunction with CsA [40].

While CsA offered significant benefits in organ transplantation, the introduction of TAC (FK506), a second CNI, marked a further enhancement in kidney transplant outcomes. Initially, TAC was employed as a salvage therapy for refractory liver allograft rejection [41]. Subsequent trials, assessing various therapeutic concentration ranges, established that a C_0_ of 5–15 ng/mL was associated with optimal efficacy and minimal toxicity for TAC [42]. In head-to-head RCTs comparing this concentration range of TAC to CsA, TAC demonstrated a substantial reduction in the incidence of acute rejection and corticosteroid-resistant rejection amongst KTR [43, 44]. A meta-analysis further supported the superiority of TAC over CsA in terms of preventing acute rejection and improving kidney allograft survival [45]. Additional benefits of TAC compared with CsA include a reduced occurrence of severe hypertension, hyperuricemia, hypercholesterolemia, hirsutism, and gum hypertrophy [46], [47], [48], [50]. As a result, the standard maintenance immunosuppression regimen transitioned to TAC, MPA, and corticosteroids at the beginning of the 21st century.

### Concern of CNI nephrotoxicity

Despite significant improvements in acute rejection rates and short-term (i.e., 1-year) allograft survival with the TAC, MPA, and corticosteroids regimen, long-term allograft survival has seen minimal improvement [51, 52]. Various proposed mechanisms contribute to these persistently poor long-term outcomes, with nephrotoxicity linked to TAC and CsA being a significant factor. Animal studies have clearly demonstrated that exposure of nephrons to CNIs results in dose-dependent constriction of afferent arterioles and acute tubular injury, leading to renal dysfunction [53]. These mechanisms involve decreased prostaglandin and nitric oxide production, as well as activation of the renin–angiotensin–aldosterone and sympathetic nervous systems [8, 9]. Additionally, CNIs can cause endothelial injury and thrombotic microangiopathy (TMA), either through vasoconstriction-related ischemia or the direct activation of pro-thrombotic factors [9, 54]. Furthermore, CNI nephrotoxicity also manifests as chronic and irreversible damage to kidney structures, including glomeruli (causing focal segmental or global glomerulosclerosis), tubulo-interstitium (characterized by interstitial fibrosis and tubular atrophy; IFTA), and vessels (resulting in medial arteriolar hyalinosis) [8, 9]. These chronic changes are believed to be one of the leading causes of long-term allograft failure [9].

Notably, a landmark study published in 2003 that analyzed 10-year surveillance biopsy results of 120 KTR, comprising 961 biopsy specimens, revealed that the prevalence of CNI nephrotoxicity was 100% at 10 years post kidney transplantation [7]. The authors concluded that CNI nephrotoxicity was a significant contributor to chronic allograft nephropathy (CAN, historically defined as chronic IFTA, vascular occlusive changes, and glomerulosclerosis), and suggested that CNIs were unsuitable for long-term immunosuppression due to these adverse effects [7]. While these statements have faced subsequent debates as will be discussed below, the transplant community has invested significant efforts in reducing the use of CNIs or minimizing their usage as much as possible to mitigate these long-term consequences, marking the beginning of the “CNI-sparing regimen” era in the early decades of the 21st century.

## CNI-sparing regimens

**Figure 1.**
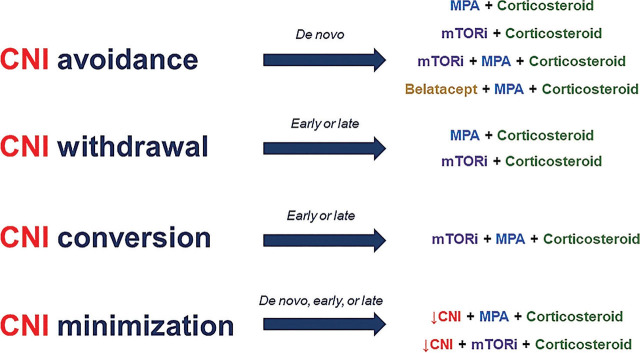
Concept of CNI-sparing strategy. The “early period” denotes interventions applied within 4–6 months after transplantation, while the “late period” refers to interventional timing after that. CNI, calcineurin inhibitor; MPA, mycophenolic acid; mTORi, mammalian target of rapamycin inhibitor.

The CNI-sparing regimens can be categorized into 4 main groups: CNI avoidance, CNI withdrawal, CNI conversion, and CNI minimization (**Figure 1**) [55], [56], [57]. CNI avoidance entails not including any CNIs from the time of transplantation. CNI withdrawal involves discontinuing CNIs from the maintenance regimen at specific time points, either early (<6 months) or late (≥6 months) after kidney transplantation. CNI conversion follows a similar strategy to CNI withdrawal, but instead of discontinuing CNIs, it substitutes them with another anti-metabolite, such as a mammalian target of rapamycin inhibitor (mTORi) or MPA, or converting to belatacept. Finally, CNI minimization uses CNIs throughout the post-transplant period but at a reduced dose to achieve a lower target therapeutic concentration (i.e., C_0_ <10 ng/mL) compared with the originally recommended concentrations in earlier eras (C_0_ 10–15 ng/mL). Numerous RCTs have explored the efficacy and safety of these CNI-sparing regimens, as extensively reviewed elsewhere [55, 56], [58], [59], [60], [61], [62].

Examples of RCTs investigating various immunosuppressive regimens are presented in **Table 1**. These RCTs are provided for illustrative purposes and do not represent all available RCTs in the corresponding categories [63], [64], [65], [66], [67], [68], [69], [70], [71], [72], [73], [74], [75], [76], [77], [78]. Several meta-analyses have been conducted to compare the benefits of these strategies with the standard CNI dose plus MPA regimen [57], [79], [80], [81], [82]. The most comprehensive and up-to-date meta-analysis of all CNI-sparing strategies was performed by Sawinski et al. [57]. The authors found that the use of CNI avoidance (mTORi combined with MPA regimen) significantly increased the risk of graft loss with a relative risk (RR) of 3.40 compared with the standard TAC and MPA regimen. CNI withdrawal, followed by only MPA or mTORi maintenance, was associated with an increased risk of acute rejection (RR 3.17 and RR 1.71, respectively). However, the use of CNI conversion to mTORi demonstrated a significant improvement in kidney allograft function without increasing the risk of acute rejection or graft loss, similar to the effect of using a reduced dose of CNI combined with mTORi or MPA compared with the standard CNI dose. Additionally, when a reduced CNI dose was combined with MPA, the risk of acute rejection was significantly decreased (RR 0.80), along with the risk of graft loss (RR 0.71), compared with the standard dose CNI with MPA regimen. From this meta-analysis, the CNI-sparing regimens that appear to have a sufficient level of evidence supporting demonstrable benefits are CNI-to-mTORi conversion and the CNI minimization strategy. Among the “no CNI regimen” strategies, including avoidance, withdrawal, and conversion, it is important to emphasize that the CNI conversion and withdrawal strategy can be influenced by large variations in the timing of intervention. This timing can range from early (within 4–6 months after transplantation) to late (after 4–6 months after transplantation). Such variation may lead to different conclusions between studies. For instance, studies with early intervention might encounter higher rates of acute rejection due to allo-sensitization after CNI removal, whereas studies with late intervention might be more likely to achieve successful conversion or withdrawal due to T cell exhaustion after a longer period post-transplantation [83, 84].

**Table 1.** Example of key randomized controlled trials in maintenance immunosuppression in kidney transplantation and an overview of their outcomes.

**CNI avoidance**	**CNI withdrawal**	**CNI conversion**	**CNI minimization**
• ELITE-Symphony [85] • ORION [72] • BENEFIT [103] • SPEISSER [65]	• CAESAR [66] • ORION [72] • Creeping Creatinine Study [64] • Rapamune Maintenance Regimen Study [63]	• CONVERT [88] • CONCEPT [68] • SMART [70] • Spare the Nephron Study [74] • ZEUS [75] • HERAKLES [77] • ASCERTAIN [73] • ELEVATE [78]	• CAESAR [66] • ELITE-Symphony [85] • OPTICEPT [67] • A2309 Study [71] • EVEREST [69] • ASSEST [76] • TRANSFORM [95]
○ **Increased risk of rejection [72, 85, 103]** ○ Some showed better GFR [103] ○ Some showed increased risk of graft loss [85] (non-belatacept study)	○ **Increased risk of rejection [66, 72]** ○ Some showed better GFR and lower viral infection rate [63, 64]	○ **Better GFR [68, 70, 73, 74, 75, 77, 78, 88]** ○ **Lower viral infection rate [70, 74, 77, 78, 88]** ○ Some showed higher rejection rate and lower cancer rate [68, 75, 78, 88]	○ **Better GFR [67, 85]** ○ Some showed lower viral infection rate [66, 71, 95]
**Limitations in interpretation** ❖ The included populations are different among studies, mostly low-to-moderate risk patients. ❖ Different tissue typing, organ allocation system, preformed anti-HLA detection. ❖ Dynamic change of outcomes: Banff classification, eGFR estimation. ❖ Different patient care: target C_0_ concentration, induction regimen and dose. ❖ Lacking of *de novo* anti-HLA detection protocol. ❖ Different follow-up duration, time of intervention (conversion and withdrawal). ❖ Different “standard” control group.			

C_0_, pre-dose concentration; CNI, calcineurin inhibitor; eGFR, estimated glomerular filtration rate; GFR, glomerular filtration rate; HLA, human leukocyte antigen

An overview of outcomes from RCTs and meta-analyses is presented in **Table 1**. However, these comparisons are limited by several factors, primarily stemming from the high heterogeneity between studies. These CNI-sparing studies were conducted between 2000 and 2018, a period marked by continuous and significant changes in the field of kidney transplantation. These changes encompassed advancements in tissue typing methods, modifications in organ allocation procedures, the detection of preformed and *de novo* antibodies against HLA, and variations in the definition of “standard” groups in different studies. Importantly, outcome measurements also varied from one study to another, with differences in approaches such as the utilization of the Banff classification and the estimation of the glomerular filtration rate (GFR), which also underwent changes over time. It is crucial to recognize the differences in study design and the implications of each study. In the following section, notable landmark RCTs for the current maintenance immunosuppressive medication regimens are discussed to provide insight into the distinctions between each regimen and their respective advantages and disadvantages. The PubMed database was searched using Medical Subject Headings (MeSH) with “kidney transplantation” and “immunosuppression therapy.” Studies were selected based on their high impact on the transplant community and guidelines, starting from the Efficacy Limiting Toxicity Elimination (ELITE)-Symphony study, which significantly contributed to the current standard maintenance regimen. A summary of these RCTs is presented in **Table 2**.

**Table 2.** Summary of modern randomized controlled trials in maintenance immunosuppression in kidney transplantation.

**Study (year)**	**KTR included**	**Type of study**	**Comparison and target C_0_ (ng/mL)**	**Allograft function (mean eGFR, mL/min)**	**Acute rejection (%)**	**Patient and graft survival**	**Other findings**
**ELITE-Symphony (NEJM 2007) [85]**	1,645	*De novo* CNI minimization *De novo* CNI avoidance	CsA (relatively higher C_0_) (150–300 for 3 months then 100–200) CsA (100–200) + daclizumab TAC (3–7) + daclizumab SRL (4–8) + daclizumab (Base: MMF + steroid)	**TAC was associated with best allograft function.** **(57, 59, 65, 57)**	**TAC was associated with lowest allograft rejection.** **(30, 27, 15, 40)**	**TAC was associated with best allograft survival. Patient survivals were not different.**	TAC was associated with lowest treatment failure rate.
**FREEDOM (AJT 2008) [112]**	337	*De novo* steroid avoidance Early steroid withdrawal	Steroid free Steroid withdrawal on D8 Standard steroid (Base: Basiliximab + CsA + MPS) (target C_2_ CsA 1,500–2,000 ng/mL during month 1, 1,300–1,700 ng/mL during month 2, 1,100–1,500 ng/mL during month 3, 900–1,300 ng/mL during months 4–6 and 800–1,000 ng/mL thereafter)	Allograft functions were similar. (56, 55, 59)	**Steroid-free regimen was associated with the highest incidence of acute rejection, followed by steroid-withdrawal, and standard steroid.** **(32, 26, 15)**	Allograft and patient survival were similar.	Steroid-free group used less anti-hyperglycemic medication. Number of KTR diagnosed with diabetes at month 12 was similar between groups.
**CONVERT (Transplantation 2009) [88]** 830 Late CNI conversion CNI continuation (CsA C_0_ 50–250 or TAC C_0_ 4–10) CNI conversion to SRL (6–120 months) (C_0_ 8–20) (Base: AZA or MMF + steroids)				Overall allograft functions were similar. (61, 64)	Acute rejections were similar. (1.5, 3.1)	Allograft and patient survival were similar.	Patients with baseline GFR >40 mL/min had significantly higher GFR after SRL conversion. SRL conversion patients with baseline UPCR >1.0 had a higher percentage of UPCR >1.0 at 24 months.
**BENEFIT (NEJM 2016) [103]**	666	*De novo* CNI avoidance	More intensive belatacept Less intensive belatacept CsA (150–300 the first month and 100–250 thereafter) (Base: Basiliximab + MMF + glucocorticoids)	**Belatacept groups were associated with better allograft function.** **(70, 72, 45)**	**Belatacept groups were associated with higher acute rejections.** **(24, 18, 11)**	**The composite of patient and graft survival was better in belatacept group (patient but not graft survival reached significant level in secondary analysis).**	*De novo* DSA was significantly lower in belatacept groups. PTLD occurred mainly in EBV negative KTR with more intensive belatacept.
**HARMONY (Lancet 2016) [113]**	587	Early steroid withdrawal	Basiliximab + standard steroid Basiliximab + steroid withdrawal on D8 ATG + steroid withdrawal on D8 (Base: advagraf + MMF) (Advagraf target C_0_ 7–12 ng/mL within the first month, 6–10 ng/mL during months 2 and 3, and 3–8 ng/mL during months 4–12)	Allograft functions were similar. (46, 47, 50)	Acute rejections were similar. (11, 11, 10)	Allograft and patient survival were similar.	Incidence of PTDM and osteoporosis were lower in steroid-withdrawal groups. Anemia was more frequent in steroid-withdrawal groups.
**TRANSFORM (JASN 2018) [95]**	2,226	*De novo* CNI minimization	EVL (C_0_ 3–8) + minimized TAC (C_0_ 4–7 ng/mL during months 0–2, 2–5 ng/mL during months 3–6, 2–4 ng/mL thereafter) or CsA (C_0_ 100–150 ng/mL, 50–100 ng/mL, and 25–50 ng/mL, respectively) MPA + TAC (C_0_ 8–12 ng/mL during months 0–2, 6–10 ng/mL during months 3–6, and 5–8 ng/mL thereafter) or CsA (C_0_ 200–300 ng/mL, 150–200 ng/mL, and 100–200 ng/mL, respectively) (Base: Basiliximab or ATG + prednisolone)	Allograft functions were similar. (53, 54)	Acute rejections were similar. (12, 9)	Allograft and patient survival were similar.	Discontinuation of study drug was more frequent in the EVL group. Infections occurred less frequently in the EVL group, mainly CMV and BKV.
**ATHENA (Kidney Int 2019) [98]**	655	*De novo* EVL vs. MPA in modern CNI exposure	EVL (C_0_ 3–8) + TAC (C_0_ 4–8 until the end of months 2 and 3–5 thereafter) EVL (C_0_ 3–8) + CsA (C_0_ 75–125 until the end of month 2 and 50–100 thereafter) MPA + TAC (C_0_ 4–8 until the end of month 2 and 3–5 thereafter) (Base: Basiliximab + prednisolone)	Noninferiority was not shown in primary analysis (eGFR margin = 7 mL/min/1.73 m^2^). (63, 61, 68)	**Acute rejections were similar between TAC groups, but was higher in CsA group.** **(7, 5, 19)**	Allograft and patient survival were similar.	Drug discontinuation was more frequent in EVL groups. Post hoc analysis showed noninferiority if eGFR margin set at 9 mL/min/1.73 m^2^. Infections were less frequent in EVL groups, mainly CMV and BKV.

Significant findings are presented in bold.

ATG, anti-thymocyte globulin; AZA, azathioprine; BENEFIT, Belatacept Evaluation of Nephroprotection and Efficacy as First-line Immunosuppression Trial; BKV, BK virus; C_0_, pre-dose concentration; C_2_, two hours-post-dose concentration; CMV, cytomegalovirus; CNI, calcineurin inhibitor; CONVERT, the Sirolimus Renal Conversion Trial; CsA, cyclosporine A; DSA, donor-specific anti-human leukocyte antigen antibody; EBV, Epstein-Barr virus; eGFR, estimated glomerular filtration rate; ELITE, Efficacy Limiting Toxicity Elimination; EVL, everolimus; GFR, glomerular filtration rate; KTR, kidney transplant recipient; MMF, mycophenolate mofetil; MPA, mycophenolic acid; MPS, mycophenolate sodium; PTDM, post-transplant diabetes mellitus; PTLD, post-transplant lymphoproliferative disorder; SRL, sirolimus; TAC, tacrolimus; TRANSFORM, Transplant Efficacy and Safety Outcomes with an Everolimus-based regimen; UPCR, urine protein-to-creatinine ratio.

## Selected clinical trials of current maintenance immunosuppression

### ELITE-Symphony study

In the current era, the pivotal RCT that holds significant importance in the realm of maintenance immunosuppression for kidney transplantation is the ELITE-Symphony study [85]. This study served as the catalyst for establishing TAC (with the current therapeutic target C_0_), MPA, and corticosteroids as the standard immunosuppressive regimen. The study encompassed 1,645 KTR from 15 countries, excluding those with a panel reactive antibody (PRA) >20% and a cold ischemic time (CIT) surpassing 30 h. KTR were randomized into 4 groups: the standard-dose CsA group (C_0_ 100–200 ng/mL), low-dose CsA group (C_0_ 50–100 ng/mL), low-dose TAC group (C_0_ 3–7 ng/mL), and low-dose sirolimus (SRL) group (C_0_ 4–8 ng/mL). The term “low-dose,” as employed in this study, was relative to the immunosuppressant dosages utilized during that period. For instance, low-dose TAC aimed at a target C_0_ of 3–7 ng/mL compared with the standard C_0_ of 10–15 ng/mL at that time. Each group received MMF (2 g/d) and prednisolone as part of the combined immunosuppressive therapy. Daclizumab was administered as the induction therapy in all groups, except for the high-dose CsA group, which did not receive any induction.

At the 12-month mark, the low-dose TAC group demonstrated superiority over the other groups in terms of the primary outcome, estimated glomerular filtration rate (eGFR), as well as secondary outcomes, including measured GFR, lower rejection rates, improved allograft survival, and reduced treatment failure. The advantages of enhanced allograft function and allograft survival persisted for at least 3 years in the subsequent extension follow-up study [86]. It is worth noting that, although the protocol specified a target C_0_ range of 3–7 ng/mL for TAC, the actual observed C_0_ in the study fell within the range of 5–10 ng/mL [87]. Consequently, CNI minimization, characterized by low-dose TAC, has since become the standard immunosuppressive regimen in kidney transplantation.

### CONVERT study

Simultaneously with the minimization strategy in the ELITE-Symphony study, the Sirolimus Renal Conversion Trial (CONVERT) investigated the CNI-conversion strategy as a means to mitigate the long-term adverse effects of CNIs [88]. In this RCT, 830 KTR within 6–120 months post-transplantation were recruited from 11 transplant centers. They were randomized to either continue with CNI (CsA C_0_ 50–250 ng/mL or TAC C_0_ 4–10 ng/mL) along with MMF or AZA and corticosteroids, or to undergo a CNI-to-SRL conversion (target SRL C_0_ of 8–20 ng/mL).

The authors observed that among KTR with a baseline eGFR >40 mL/min (calculated using the Nankivell formula) and a baseline urine protein-to-creatinine ratio (UPCR) of ≤0.11, who underwent conversion to SRL, the mean eGFR was significantly higher than in the CNI-continuation group at both 12 months and 24 months after conversion. There were no significant differences in acute rejection rates, allograft survival, or patient survival between the 2 groups. However, a higher percentage of KTR in the SRL conversion group had to discontinue the treatment compared with the CNI-continuation group (15.7% vs. 9.5%) due to intolerable adverse effects of SRL including anemia, peripheral edema, fever, skin rash, thrombocytopenia, and leukopenia. Enrollment of KTR with a baseline eGFR of 20–40 mL/min was halted by the Drug Safety Monitoring Board due to a significantly higher number of KTR in the SRL conversion group experiencing acute rejection, graft loss, or mortality compared with the CNI-continuation group.

The results from the CONVERT study were consistent with previous observational studies, which found that lower baseline proteinuria was predictive of successful responders (those with stable or improved kidney allograft function) amongst KTR undergoing CNI-to-SRL conversion [89]. Consequently, the Kidney Disease: Improving Global Outcomes (KDIGO) Transplant Work Group recommended the replacement of CNI with mTORi in KTR experiencing CNI nephrotoxicity, provided their eGFR >40 mL/min/1.73 m_2_ and their UPCR is <500 mg/g creatinine [90].

### TRANSFORM study

After a decade of utilizing low-dose TAC (C_0_ 5–10 ng/mL) in combination with an MPA/corticosteroids regimen, there has been a growing trend towards using even lower doses of CNIs (a TAC C_0_ <5 ng/mL) in conjunction with mTORi, to further mitigate the adverse effects of CNI nephrotoxicity [55, 58, 80]. Evidence has indicated that replacing MPA with mTORi provides additional benefits, including a reduced incidence of post-transplant malignancies, particularly skin cancer and Kaposi's sarcoma, as well as a decreased risk of cytomegalovirus (CMV) and BK virus (BKV) infections [91], [92], [93], [94]. The Transplant Efficacy and Safety Outcomes with an Everolimus-based regimen (TRANSFORM) study is the largest RCT ever conducted in kidney transplantation, involving 2,226 KTR from 186 transplant centers [95]. The primary outcome aimed to compare the composite of biopsy-proven acute rejection and eGFR <50 mL/min/1.73 m^2^ at 12 months between KTR randomized to either the standard CNI with MPA group or the *de novo* CNI minimization with everolimus (EVL) group. The standard CNI with MPA group targeted C_0_ TAC at 5–8 ng/mL or CsA at 200–300 ng/mL, while the CNI minimization group aimed for C_0_ TAC at 2–4 ng/mL or CsA at 25–50 ng/mL combined with EVL at a C_0_ of 3–8 ng/mL during the maintenance period. Both groups received basiliximab as an induction therapy. The EVL group was found to be non-inferior to the MPA group for the primary endpoint at 12 months post-transplantation. There were no differences in terms of *de novo* donor-specific anti-HLA antibody (DSA) development, although this was assessed locally by available centers. However, the EVL group demonstrated a significantly lower incidence of CMV and BKV infections compared with the MPA group. The discontinuation rate was higher in the EVL group. In a 2-year follow-up study, EVL continued to be non-inferior to the MPA group [96]. This represents the first study to demonstrate the non-inferiority of *de novo* mTORi use, which allows for further reduction of CNI exposure compared with the current standard CNI dose with MPA. However, the long-term outcomes, particularly concerning DSA and ABMR, remain to be determined. In elderly KTR, who are less likely to reject their kidney allografts but are more likely to have infectious complications after transplantation, the Open Label Multicenter Randomized Trial Comparing Standard Immunosuppression with TAC and MMF with a Low Exposure TAC Regimen in Combination with EVL in *De novo* Renal Transplantation in Elderly Patients (OPTIMIZE) trial is currently running in the Netherlands to investigate the use of CNI-minimization in this specific population [97].

### ATHENA study

During approximately the same period as the TRANSFORM study, another group of investigators designed the ATHENA study with the aim of evaluating the efficacy of reduced-dose TAC (TAC C_0_ 3–5 ng/mL) in combination with EVL (C_0_ 3–8 ng/mL, identical to the EVL group in the TRANSFORM study) vs. reduced-dose TAC (C_0_ 3–5 ng/mL) in combination with MPA [98]. The ATHENA study sought to employ lower C_0_ levels of TAC in the MPA group compared with the TRANSFORM study (i.e., comparing the regimen containing EVL vs. MPA with similar reduced-dose TAC). The primary outcome of the ATHENA study was the eGFR at 12 months post-transplantation.

A total of 665 KTR from 27 centers were included in the ATHENA study. Although the study protocol stipulated that the TAC C_0_ should fall between 3 ng/mL and 5 ng/mL, the actual results revealed that both the EVL and MPA groups had significantly higher TAC C_0_ than the planned maximum throughout the study. At month 12 of the study, the TAC C_0_ for the EVL group averaged 6.0 ± 2.2 ng/mL, while in the MPA group, it was 5.9 ± 2.1 ng/mL. The prespecified non-inferiority margin for eGFR, set at 7 mL/min/1.73 m^2^, was not statistically achieved. However, a *post hoc* non-inferiority margin of 9 mL/min/1.73 m^2^ demonstrated non-inferiority between the EVL and the MPA groups. The authors discussed that the failure to show non-inferiority with the prespecified margin might have been due to insufficient differences between the treatment groups or that the TAC exposure in the groups was too high. Nevertheless, the EVL group was associated with a lower incidence of CMV and BKV infections, reaffirming the benefits of using mTORi, as also demonstrated in the TRANSFORM study [95]. Neither the ATHENA study nor the TRANSFORM study performed TDM of MPA, limiting the assessment of concentration-dependent efficacy and toxicity in the MPA group [99].

### Belatacept study

Alongside the CNI minimization and CNI conversion strategies discussed earlier, CNI avoidance using belatacept has been extensively studied with impressive results [100]. Belatacept is a fusion protein comprising the Fc fragment of IgG1 and the extracellular domain of the cytotoxic T-lymphocyte-associated protein 4 (CTLA-4) [101]. Its primary function is to inhibit the costimulatory signal between CD80/86 and CD28 in the T cell activation process. The Belatacept Evaluation of Nephroprotection and Efficacy as First-line Immunosuppression Trial (BENEFIT) randomized 666 *de novo* KTR, excluding those with PRA >50% and a CIT >24 h, into 1 of 3 groups [102]. The first and second groups received either more intensive or less intensive belatacept in combination with MMF and glucocorticoids. The more intensive group was administered 11 doses of 10 mg/kg of belatacept intravenously in the first 6 months, followed by 5 mg/kg every 4 weeks, while the less intensive belatacept group received 6 doses of 10 mg/kg in the first 3 months, followed by 5 mg/kg every 4 weeks thereafter. The third group served as the standard comparator at the time of the study's design, involving CsA aiming for a C_0_ of 100–250 ng/mL and MMF/corticosteroids during the maintenance phase. The BENEFIT study is a modern RCT with one of the longest follow-up times of an immunosuppression regimen ever reported, with results spanning up to 7 years after transplantation [103]. At 7 years, both more intensive and less intensive belatacept regimens exhibited a 43% reduction in the risk of death or graft loss. Additionally, eGFR in the belatacept groups was significantly superior to that in the CsA group throughout the study period. KTR receiving belatacept also developed significantly fewer *de novo* DSA than those on CsA. While the rates of biopsy-proven acute rejection were higher in the belatacept groups, these rejections were considered manageable and did not adversely affect the long-term outcomes of the recipients. Importantly, the incidence of post-transplant lymphoproliferative disorder (PTLD) was found to be high among KTR with negative Epstein-Barr virus (EBV) serology who received belatacept. This led the US Food and Drug Administration (FDA) to contraindicate the use of belatacept for recipients with negative EBV serology [101]. It is worth emphasizing that the comparator in the BENEFIT study was CsA, which is not a standard CNI in the current era.

One RCT investigated the efficacy of *de novo* belatacept (n = 104) compared with TAC (n = 105) in an early steroid withdrawal regimen, with both groups receiving anti-thymocyte globulin (ATG) and MPA [104]. The 2-year outcomes revealed comparable patient and allograft survival. Similar to the BENEFIT study, KTR with belatacept also had a higher incidence of biopsy-proven acute rejection compared with the TAC group (25% vs. 7%), although the eGFR in the belatacept group was significantly better than in the TAC group. In another smaller RCT, KTR receiving *de novo* belatacept (n = 20) were compared with those receiving TAC (n = 20) in the context of basiliximab induction and an MMF/prednisolone maintenance regimen. The study revealed that the belatacept group had a significantly higher incidence of acute T-cell mediated rejection (TCMR), which was also more severe compared with the TAC group. This resulted in a trend toward a higher graft loss rate at 1-year in the belatacept group (*P* = 0.08) [105]. Although patient survival did not differ between the groups, this pilot study confirmed that belatacept may lead to a higher incidence of acute TCMR when compared with a TAC-based maintenance regimen.

Currently, belatacept is employed as an alternative to CNIs, particularly in cases where CNIs are contraindicated, such as in individuals who have experienced CNI-associated TMA or posterior reversible encephalopathy syndrome (PRES) [106]. However, the requirement for intravenous infusion and the associated infusion-related reactions have hindered its widespread use. Another strategy involving belatacept use is late conversion (after 6 months) from TAC to mitigate the risk of TCMR in the early period after transplantation while preserving graft function. This strategy has shown that kidney allograft function improves after belatacept conversion without an increased risk of rejection [107], [108], [109]. Additionally, belatacept conversion might benefit patients with ABMR, as indicated in case series and cohorts that have demonstrated stabilization of allograft function in KTR with ABMR [110, 111]. However, an RCT is required to investigate this strategy further.

### Steroid withdrawal studies

Two landmark trials have investigated steroid withdrawal regimens in kidney transplantation. The FREEDOM study involved 337 KTR from 40 sites and randomized them into 3 groups: the steroid-free group, the early steroid withdrawal group (withdrawal after 7 d post-transplantation), and the standard steroid regimen, in which prednisolone was tapered to 5–10 mg/d after 2 months [112]. All KTR received basiliximab, CsA, and MPA. At 1-year post-transplantation, there was no difference in terms of eGFR, death, or graft loss between the early steroid withdrawal group and the standard steroid group, despite the increased incidence of acute rejection (26.1% in the steroid withdrawal group vs.14.7% in the standard steroid group). However, the composite of acute rejection, death, or graft loss was significantly higher in the steroid-free group compared with the standard steroid group (36% vs. 19%). The steroid withdrawal group exhibited a lower incidence of increased body mass index (BMI), required fewer lipid-lowering medications, and had lower triglyceride levels compared with the standard steroid group.

Another recent RCT exploring steroid withdrawal in kidney transplantation was the HARMONY trial [113]. A total of 587 KTR from 21 transplant centers were included in 1 of 3 groups: the standard group (basiliximab, TAC, MPA, prednisolone tapered to 2.5–5.0 mg/d after 3 months), the basiliximab with steroid withdrawal group (same as the standard group but with prednisolone stopped after 7 d), and the ATG with steroid withdrawal group (similar to the other steroid withdrawal group but using ATG instead of basiliximab). The included KTR were considered to have a low-to-moderate immunological risk, and those with PRA >30% were excluded. There was no difference in the primary endpoint of biopsy-proven acute rejection at 12 months. However, the steroid withdrawal groups showed benefits in terms of a lower incidence of post-transplant diabetes mellitus (PTDM) compared with the standard group. The steroid withdrawal groups also had a lower incidence of osteoporosis.

Based on the results from these steroid withdrawal RCTs, it is recommended that if a steroid withdrawal regimen is planned, it should be initiated within the first week after transplantation to decrease the metabolic adverse effects while maintaining an adequate level of immunosuppression [90]. Previous studies have shown that steroid withdrawal regimens are associated with a higher incidence of recurrent glomerular diseases, particularly IgA nephropathy [114], [115], [116], while other studies did not demonstrate an increased risk of recurrent glomerular disease [117, 118]. However, this information is limited by the retrospective design of these studies, resulting in mixed differences in the timing of steroid withdrawal (early vs. late) and the effects of other immunosuppressive medications used in the regimens during different eras. Additionally, the risk of recurrent IgA nephropathy does not depend solely on the steroid regimen but rather on other factors including age, time to ESRD, and dialysis vintage [119]. Therefore, the risk of recurrent IgA nephropathy in KTR with steroid withdrawal regimens may indeed exist. However, the extent of this burden in the current immunosuppression scheme necessitates further RCTs specifically examining this outcome with a sufficient follow-up period.

## Looking back at CNI nephrotoxicity: Is it really a cause of great loss?

Nephrotoxicity arising from CNIs is one of the most concerning complications after kidney transplantation, and it is widely believed that CNI nephrotoxicity plays a significant role in allograft failure [7, 9, 10]. CNI nephrotoxicity is also a significant contributor to chronic kidney disease in the native kidneys following non-kidney solid organ transplantation [120], [121], [122]. This has prompted extensive efforts to reduce CNI exposure, as mentioned above. However, there is evidence suggesting that the association between CNI nephrotoxicity and negative outcomes may not be as clear-cut as initially perceived.

First, there are no histological lesions that are pathognomonic or specific for chronic CNI nephrotoxicity. In studies investigating chronic lesions in kidney allograft biopsies, none of the Banff categories was found exclusively in KTR who received CNIs but absent in those on non-CNI regimens [123, 124]. These chronic lesions include tubular atrophy, interstitial fibrosis, vascular fibrous intimal thickening, or arteriolar hyalinosis (either intimal or medial). Allograft biopsies from KTR who have never received CNIs also exhibited these histological lesions, albeit at a lower incidence than in KTR who had received CNIs [124]. This indicates that CNIs might contribute to the development of chronicity lesions through various pathways, but that they lack specificity.

Second, while CNI nephrotoxicity was long considered a significant factor in allograft loss, the reality appears different. In a study that followed 1,317 KTR for a mean follow-up of 50 ± 32 months, 153 grafts (12%) were lost due to graft failure after censoring for death [125]. Among these graft failures, only 1 case of kidney allograft was attributed to CNI nephrotoxicity, while the majority of grafts within the IFTA category were lost due to conditions such as polyomavirus nephropathy, recurrent pyelonephritis, TCMR, ABMR, and poor allograft quality [125]. Infections in kidney allografts and the baseline pathology of the donor kidney can lead to IFTA in subsequent biopsies [126, 127]. Another study conducted a survival analysis after for-cause allograft biopsies, and demonstrated that kidney allografts diagnosed with CNI nephrotoxicity did not exhibit inferior allograft survival when compared with allografts with other diagnoses [128].

Arguably, the most significant evidence diminishing the importance of CNI nephrotoxicity with regard to long-term outcomes is the increasing recognition of ABMR and DSA as the leading cause of graft failure [129], [130], [131]. In contrast to earlier studies when the possibility to diagnose ABMR and the DSA detection were limited, contemporary studies with well-defined criteria for the diagnosis of ABMR have identified it as the primary cause of long-term allograft loss [131, 132]. Chronic ABMR and TCMR contribute to allograft IFTA, arteriolosclerosis, and arteriosclerosis [133], [134], [135], [136]. Furthermore, investigations into gene expression and molecular phenotypes of allograft biopsies have unveiled that the extent of inflammation in areas of IFTA or within the scarred regions of the allograft, sharing molecular phenotypes with acute rejection, has a substantial impact on long-term outcomes and graft loss [137, 138]. Conversely, allografts displaying only phenotypic features of CNI nephrotoxicity (e.g., arteriolar hyalinosis or the ah score in Banff classification) in the absence of inflammation were not associated with decreased allograft survival, as shown in the Deterioration of Kidney Allograft Function (DeKAF) study [132].

It is undeniable that chronic CNI nephrotoxicity exists. It may be related to multiple factors, including the imbalance between intra-allograft and systemic CNI exposure and clearance [139, 140]. However, the concept of the major causes of long-term allograft loss has shifted away from CNI nephrotoxicity to chronic unopposed inflammation in kidney allografts, either in the form of chronic ABMR or TCMR, with immunosuppressive medication non-adherence being an important risk factor [141], [142], [143]. The process of developing IFTA and arteriolosclerosis in kidney allografts is complex and not fully understood [10], potentially involving multifactorial causes. However, only a very small proportion of graft loss is attributed to IFTA without inflammation, as observed in CNI nephrotoxicity [132]. Clinicians should be aware that when late kidney allograft dysfunction presents with a histological picture of IFTA or arteriolosclerosis, chronic immune-mediated injury should be ruled out first before considering a CNI-sparing regimen. Gene expression profiles of allograft biopsy samples might aid in determining the nature of IFTA, whether inflammation is present or not [137]. Reflecting on the landmark study from 2003 [7], which highlighted a 100% prevalence of CNI nephrotoxicity at 10 years and served as a catalyst for the initiation of CNI-sparing regimens, many debates have emerged in the contemporary era. Significantly, chronic CNI nephrotoxicity lacks specific histological lesions, and attributing allograft function decline solely to CNI nephrotoxicity remains inconclusive, primarily due to the absence of DSA testing.

## Perspective and conclusion

Numerous contemporary RCTs have been conducted to offer various options for maintenance immunosuppression in kidney transplantation, beyond the conventional TAC/MPA/corticosteroid regimen. The initial goal of reducing CNIs to the lowest possible exposure to prevent allograft loss from CNI nephrotoxicity has gradually evolved. This transformation is driven by current evidence, which does not strongly support the notion that chronic CNI nephrotoxicity is the primary cause of graft loss [142, 143]. Instead, alternative explanations, particularly chronic inflammation resulting from unopposed immune processes, play a significant role in shaping the long-term outcomes of allografts.

As a result, the standard regimen of TAC/MPA/corticosteroids remains the cornerstone of immunosuppression for kidney transplantation, as it has consistently demonstrated superior outcomes in terms of reducing allograft rejection and improving allograft survival [144]. Nonetheless, CNI-sparing strategies still hold value, particularly for KTR with low-to-moderate immunological risk but a heightened risk for viral infections or malignancies. KTR who cannot tolerate CNIs, such as those with a history of CNI-induced TMA, are also potential candidates for CNI-sparing regimens. Most modern CNI-sparing strategies, like CNI minimization in the TRANSFORM study, have exhibited excellent and comparable short-term outcomes when compared with the standard regimen. However, comprehensive long-term data, especially information regarding the development of *de novo* DSA and chronic ABMR and/or TCMR, are still needed to determine whether these alternative strategies are equivalent to or possibly superior to the standard regimen. TDM of TAC, mTORi, and MPA is also crucial for enhancing efficacy and minimizing adverse effects of immunosuppressive medications [145]. Moreover, clinicians should stay updated on the latest developments of novel immunosuppressants for treating rejection, which may eventually replace current maintenance regimens in the future [146, 147].

In conclusion, CNI nephrotoxicity is not a major contribution to graft loss and the standard TAC with MPA regimen remains the preferred choice. CNI-sparing approaches are considered as alternative options but necessitate additional long-term data for validation.
